# The Durability of Mortar Containing Alkali Activated Fly Ash-Based Lightweight Aggregate

**DOI:** 10.3390/ma14133741

**Published:** 2021-07-04

**Authors:** Puput Risdanareni, Philip Van den Heede, Jianyun Wang, Nele De Belie

**Affiliations:** 1 Magnel-Vandepitte Laboratory for Structural Engineering and Building Materials, Ghent University, Technologiepark Zwijnaarde 60, B-9052 Ghent, Belgium; philip.vandenheede@ugent.be; 2 Department of Civil Engineering, Faculty of Engineering, State University of Malang, Semarang Street 5, Malang 65145, Indonesia; 3 Department of Civil Engineering, Xi’an Jiaotong University, Xianning West Road 28, Xi’an 710049, China; jianyun.wang@xjtu.edu.cn

**Keywords:** fly ash, alkali activated lightweight aggregate, carbonation resistance, chloride migration, capillary water uptake, ITZ

## Abstract

Beneficiating fly ash as valuable construction material such as artificial lightweight aggregate (LWA) could be an alternative solution to increase the utilization of the industrial by-product. However, generally, LWA is characterized by high porosity and a related high water absorption, which on the one hand allows production of lightweight mortar, but on the other hand can affect its performance. Thus, in this research, the durability performance of mortar composed with alkali-activated fly ash-based LWA, and commercial expanded clay (EC) LWA was investigated. The fly ash LWA was prepared in a pan granulator, with a 6-molar solution of NaOH mixed with Na_2_SiO_3_ in a Na_2_SiO_3_/NaOH weight ratio of 1.5 being used as activator (FA 6M LWA). The results revealed that mortar containing FA 6M LWA had equivalent mechanical strength with mortar containing EC LWA. The mortar containing FA 6M LWA had comparable capillary water uptake and chloride migration resistance with the reference and EC LWA mortar. Furthermore, the addition of FA 6M LWA was proven to enhance the carbonation resistance in the resulting mortar, due to the denser interfacial transition zone (ITZ) of mortar with LWA.

## 1. Introduction

In the past decade, recycling industrial by-products or waste into more valuable materials has become the concern of many researchers around the world. It is reported that in 2015, the global production of coal combustion products (CCP) such fly ash and bottom ash was 780 metric tons, and was expected to increase by 1.6% annually [1]. Although 50% of those ashes are already being used as cement replacement, there are still abundant amounts of fly ash considered as waste [2]. Thus, the idea of beneficiating fly ash into artificial lightweight aggregate becomes attractive as a solution to up-scale the consumption of this industrial by-product [3,4,5,6,7].

The cold bonding method, in which granules are formed by mixing a supplementary cementitious material with cement or an alkali activator, has been chosen previously for producing LWA, as it requires less energy compared to the sintering method [8,9]. However, LWA produced with the cold bonding method mostly suffers from high water absorption [4,10]. Moreover, most researchers are still using cement as a binder when producing LWA. Less literature can be found on alkali activated binding, which is more environmentally friendly as it does not use Portland clinker, which has a high carbon footprint. In earlier research, it was found that this binding system enhanced the properties of LWA and did not require a NaOH concentration higher than 10M to be activated [5,6,11]. In our own previous study it was established that a NaOH concentration of 6M is sufficient to produce fly ash-based LWA [12]. The lower concentration avoids a fast setting of the paste before the proper LWA granules have formed [6,12]. 

A previous study on incorporating commercial LWA into mortar mixtures revealed that even though the resulting mortar becomes more permeable for water or gas to penetrate into the mortar compared to those using river sand, it has better resistance against chloride penetration [13,14,15,16]. The reduction of pore percolation at the interfacial transition zone (ITZ) and the penetration of hydration products into the LWA’s open pores was the reason for this improvement [13]. However, most researchers only focused on the durability of lightweight concrete incorporating commercial LWA aggregate. Limited studies could be found on the performance of mortar incorporating pelletized alkali activated artificial aggregate.

In order to understand the behavior of mortar incorporating alkali activated fly ash-based LWA, tests such as compressive strength, water absorption under vacuum, bulk density, capillary water uptake, chloride migration, and carbonation resistance were performed. The ITZ morphology between LWA and cement paste was also investigated. In the end, the results obtained were compared to mortar incorporating expanded clay LWA and natural river sand in order to figure out the potential of employing alkali activated fly ash-based LWA in the construction industry. 

## 2. Materials and Methods

### 2.1. Raw Materials and Its Characteristic

**Figure 1:**
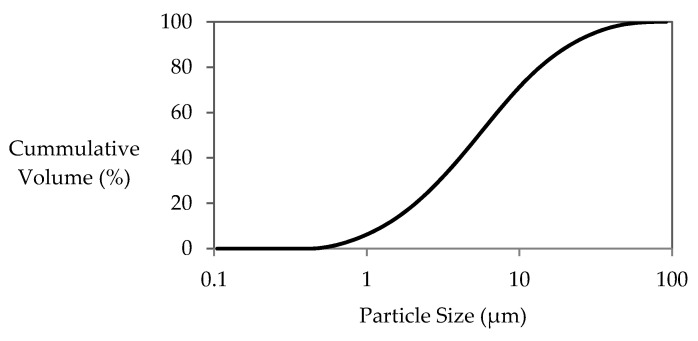
Particle Size Distribution of Fly Ash.

Fly ash (FA) class F from a Dutch power plant was used as a precursor in manufacturing artificial lightweight aggregate (LWA). The chemical composition of the fly ash, measured by X-Ray Fluorescence (XRF), is presented in Table 1. The particle size distribution of fly ash particles determined with a laser diffraction apparatus (Malvern Mastersizer 2000) is displayed in Figure 1. The absorption index and refractive index of fly ash were 0.1 and 1.67, respectively. The obscuration level was kept between 12 and 15. The dispersant (iso propanol) and the sample were stirred with the speed of 800 rpm during the measurement. The d_50_ values were obtained by averaging 6 measurement values.

Alkali activation was chosen as a binding method, while a mix of NaOH with a concentration of 6 molars and Na_2_SiO_3_ was employed as an alkaline activator. NaOH solution was obtained by diluting 240 g of NaOH powder with a purity of 98 % (VWR Belgium) in 1 L distilled water one day before use. A liquid form of Na_2_SiO_3_ was applied, which contained 26.3% silica, 7.9% sodium oxide, and 65.8% water. Furthermore, the Na_2_SiO_3_ and NaOH were mixed with a weight ratio of 1.5.

A standard river sand 0/4 was used as fine aggregate in mortar production. While a ready to use LWA generated from sintered expanded clay (EC LWA) with a fraction of 0/4 from Argex NV Belgium was studied for comparison in order to see the feasibility of applying the fly ash-based LWA in the construction industry. 

### 2.2. The Production Process and the Properties of Fly Ash Based LWA

**Figure 2:**
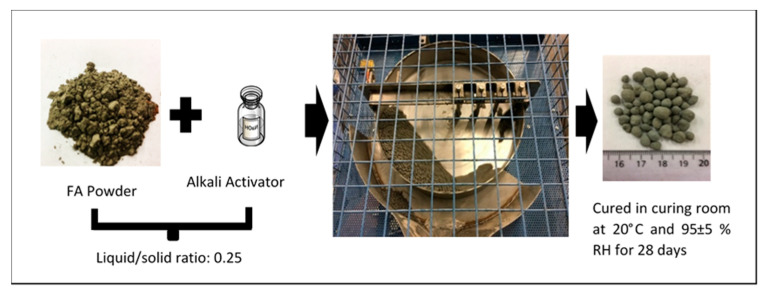
Illustration of the LWA Production.

Fly ash based LWA was produced with the agglomeration technique using a pan granulator with the diameter of 500 mm and the depth of 95 mm. The slope of the pan granulator was set at 48° while the speed was set at 60 rpm. This set up was chosen based on previous research by Baykal et al., which reported optimal LWA properties for these parameter settings [7]. The liquid/solid ratio of 0.25 was applied based on previous research [3,10,17]. The LWA production was initiated by adding fly ash powder into the pan, followed by spraying the required amount of mixed alkali activator continuously. During that time, the fly ash powder will bond with the alkali activator and form granules. After being rotated for approximately 20 min, due to the gravity movement, the compacted pellets will fall out through the side of the pan (Figure 2). Later on, the wet granules were dried in a curing chamber with a temperature of 20 ± 2 °C and humidity of 95 ± 5% for 24 h. Then, the dried LWAs were sieved with a 2 mm sieve in order to remove dust and the finer fraction of LWA. Finally, dried sieved LWAs were moved into a sealed plastic bag and further cured in the same chamber for 28 days before being applied into a mortar.

**Figure 3:**
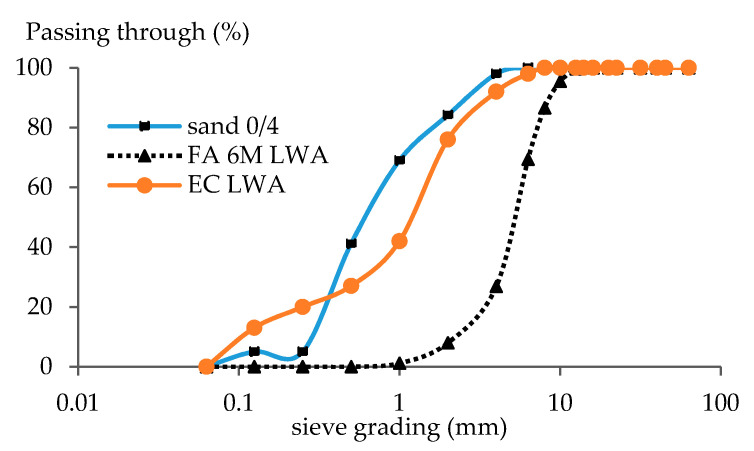
Particle Size Distribution of The Aggregates.

**Figure 4:**
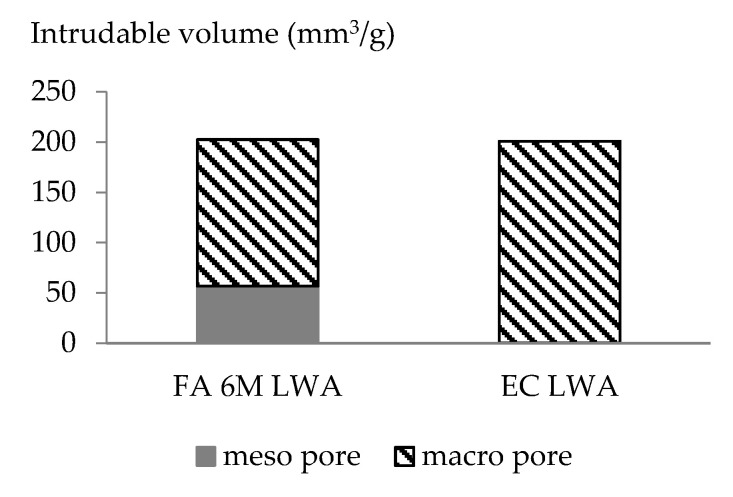
Pore characterization of EC LWA and FA 6M LWA.

The physical properties of LWA such as apparent density, oven dried density, saturated surface dry (SSD) density, and water absorption over 24 h were tested according to the NBN EN 1097-6 standard and the values are displayed in Table 2. The particle size distribution of aggregates was determined in accordance with the Belgian standard NBN EN 12620 and the values are presented in Figure 3. The IUPAC guidelines were used to characterize the LWA’s pores derived from mercury intrusion porosimetry (MIP) (Figure 4). Mesopores are defined as having a size of 2–50 nm, while macropores have a diameter above 50 nm [18]. More detailed data on the pore structure of LWA using MIP were reported in a previous article [19]. The other properties of fly-ash based LWA such crushing strength resistance and mineralogy has been studied in previous research [12,19]

### 2.3. Mortar Production

All mortar specimens were produced by following the guidelines from the -NBN EN 196-1 standard. Prisms with a dimension of 40 × 40 × 160 mm^3^ were used for testing compressive strength, capillary water absorption and carbonation. Cylinders with a diameter of 100 mm and a height of 200 mm were used for the water absorption under vacuum and the chloride migration test. 

The material needed to manufacture three mortar prisms is presented in Table 3. Cement type I 52.5 from Holcim was used as binder while river sand with fraction of 0/4 was used as fine aggregate. The volume of river sand with a size of 2–4 mm (see Figure 3) was replaced with the LWA. The particle sizes to be replaced with LWA were chosen in the range of 2–4 mm, as LWA with granules lower than 2 mm are more fragile. This implies that 16% of the total volume of 0/4 mm sand was replaced with LWA to maintain the distribution of fine aggregates. However, some literature mentions a higher replacement rate of LWA up to 21% by volume, and no workability issues were reported [5]. The corresponding weight ratio was calculated based on the apparent density. In order to avoid workability issues during mortar production due to the high water absorption of LWA, a certain amount of extra water (entrained water) was added based on LWA’s water absorption value for the LWA to reach saturated surface dry (SSD) condition. Finally, the total amount of fine aggregate (LWA SSD condition and sand 0–2) in the mixture was kept at 1350 g/batch. 

After being cast, all mortar specimens were covered with plastic film to avoid evaporation and were cured in a curing chamber which has a temperature of 20 ± 2 °C and humidity of 95 ± 5% for 24 h. After 24 h, the specimens were demolded and were left in the same chamber for 28 days before being tested.

### 2.4. Physical Properties of Resulting Mortar

The tested physical properties of the resulting mortar were compressive strength, water absorption, apparent density, and the amount of open porosity. The compressive strength of the mortar was determined as an average value of three replicate samples according to the NBN EN 196-1 standard. The test was conducted on prismatic specimens, which had been cured for 28 days in a room with a temperature of 20 ± 2 °C and humidity of 95 ± 5%.

The water absorption (WA), apparent density (AD), and the amount of open porosity (OP) of mortar were determined on three cylindrical specimens with a diameter of 100 mm and a height of 50 mm by following the guidelines from EN 1936 standard. These cylinders were obtained by cutting the cast cylinder specimens, which had a diameter of 100 mm and a height of 200 mm into 3 slices. The top 25 mm and bottom 25 mm of the cylinder were removed.

First, the samples were left in a sealed desiccator. Vacuum pressure was then applied for 2 h. After that, water was let into the desiccator until all the samples were fully immersed. After being immersed in the water for 24 h, samples were taken out and weighed. This weight was denoted as a saturated mass in the air (*m_sa_*). Later on, samples were weighed in water. This mass was denoted as a saturated mass in water (*m_sw_*). After that, samples were dried in the oven with a temperature of 105 °C until constant mass was reached. The weight of the dried samples was then denoted as the dry mass (*m_d_*). Finally, the water absorption (*WA*), the apparent density (*AD*), and the open porosity (*OP*) were calculated using Equations (1)–(3), respectively. Finally, the water absorption, apparent density and open porosity are shown as average value of three replicate samples, with the standard deviation on the mean presented by the error bars.

(1) $$WA=\frac{m_{sa}-m_{d}}{m_{d}}$$

(2) $$AD=\frac{m_{d}}{m_{sw}-m_{d}}x\;\rho_{water}$$

(3) $$OP=\frac{m_{sa}-m_{d}}{m_{sa}-m_{sw}}$$

where *ρ_water_* is the density of water at 20 °C which is 998 kg/m^3^.

### 2.5. Chloride Migration

The chloride migration test was conducted on six mortar cylinders, which have a diameter of 100 mm and a height of 50 mm. This test was performed according to the NT Build 492 practical guidelines. 

First, samples were put in a sealed desiccator, and vacuum pressure was introduced and was maintained for 3 h. Under the vacuum condition, Ca(OH)_2_ solution with a concentration of 4 g/L was added until all the specimens were immersed. After that, the vacuum condition was maintained for another hour. Finally, the vacuum was released, and the specimens were left immersed for 18 ± 2 h. 

Afterwards, the samples were fixed into rubber sleeves with an anolyte solution (3M NaOH) poured on the top. The bottom parts of the samples were in contact with the catholyte solution (NaCl 10%). An external electrical potential of 30 V was applied axially across the specimens to force the chloride ions to migrate into the specimen. After 24 h, the samples were removed and split axially. Later on, a 0.1 N silver nitrate solution was sprayed on the split surface of the sample. After a white layer of precipitated silver nitrate became clearly visible, the penetration depth was measured with intervals of 10 mm. 

Finally, the chloride migration coefficient (*D_nssm_*) was calculated using Equation (4).

(4) $$D_{nssm}=\frac{0.0239\left(273+T\right)L}{\left(U-2\right)t}\left(x_{d}-0.0238\sqrt{\frac{\left(273+T\right)L\;X_{d}}{U-2}}\right)$$

where *D_nssm_* is the non-steady migration coefficient (10^−12^ m^2^/s), *U* is the absolute voltage applied (V), T is an average value of initial and final temperature in anode solution (°C), *L* is the thickness of the sample (mm), *X_d_* is an average value of penetration depth (mm) and t is test duration. 

### 2.6. Capillary Water Uptake

**Figure 5:**
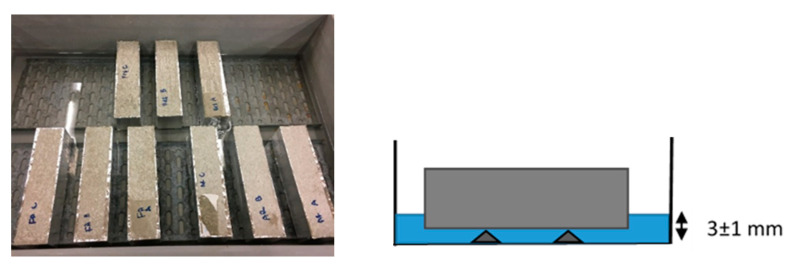
Capillary water uptake test set up.

The capillary water uptake test was performed on three mortar prisms with a dimension of 40 × 40 × 160 mm^3^, following the guidelines from EN 1015-10. Firstly, 28 days old mortar samples were further cured in a conditioned room with a temperature of 20 ± 2 °C and relative humidity of 60 ± 5% for another 28 days. One day before the test, the side surfaces of the prisms were fully covered with aluminum tape and the samples were weighed. Later on, the mortar prisms were put on metal supports in a water-filled container with an immersion depth of 3 ± 1 mm (Figure 5). The weight change of the samples was monitored after 0.5 h, 1 h, 2 h, 3 h, 4 h, 5 h, 6 h, 24 h, and then every 24 h for one week.

### 2.7. Carbonation Resistance

The carbonation test was performed on prismatic mortar samples in accordance with Belgian standard NBN B15-100. The 28 days old samples were moved from the curing room with a temperature of 20 ± 2 °C and humidity of 95 ± 5% to a conditioned room with lower humidity (60 ± 5%) for another 28 days. After that, 3 sides of the samples were covered with aluminum tape, leaving the bottom surface exposed. Furthermore, the samples were placed in the carbonation chamber at 1% CO_2_, a temperature of 20 °C and a relative humidity of 60% with the not-covered surfaces upwards. After being exposed for 4, 12, and 16 weeks, the mortar bars were split perpendicular to the exposed surface and the split surfaces were sprayed with 1% phenolphthalein solution, for colorimetric carbonation assessment. 

### 2.8. Morphology of ITZ

#### 2.8.1. Sample Preparation

The mortar samples were cut to slices with a thickness of 2 mm using a diamond saw cutter. These samples were broken until they fit in a cylindrical mold with a diameter of 30 mm. These pieces of mortar then were immersed in methanol to stop the hydration. Next, they were dried in an oven at 40°C until they reached constant weight. 

The next step was sample impregnation with epoxy. The samples were subjected to a vacuum pressure (0.8 bar) for 2 h, followed by adding the epoxy solution. The impregnated samples were then left in an oven at 40 °C for 24 h until the epoxy had dried.

Finally, the samples were polished with a SiC paper with a grit size of 320, 500, 1200, and 2400 (coarser to finer) for approximately 1.5 min each, followed by polishing with diamond paste with a grit size of 3 and 1 µm for 4 min. The polished samples were dried in the oven at 40 °C for 24 h. The carbon coating was then applied as the final stage of sample preparation. 

#### 2.8.2. Image Acquisition and Elemental Analysis

The back scattered electron (BSE) images were taken with a Jeol JSM-7600F Field Emission Scanning Electron Microscope (FESEM) equipped with AZtecEnergy software from Oxford Instruments for EDX mapping. EDX mapping was performed for Ca, Si, Fe, and Al elements.

## 3. Results and Discussion

### 3.1. Physical Properties of Resulting Mortar

**Figure 6:**
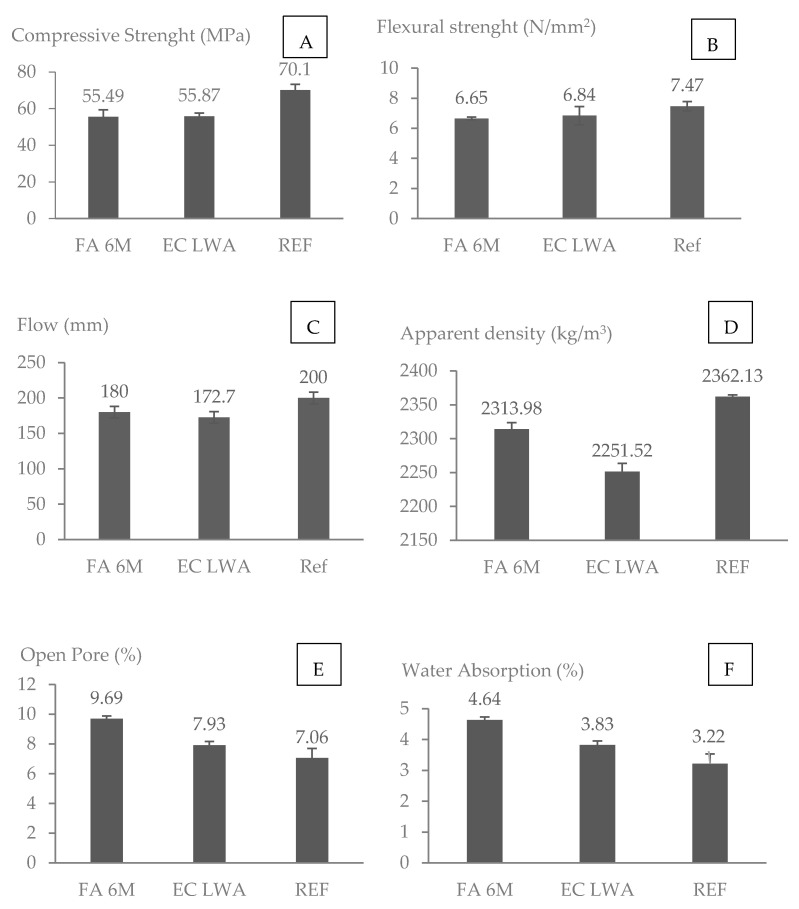
Physical properties of resulting mortar: (**A**) the compressive strength; (**B**) Flexural Strength; (**C**) Workability of Fresh Mortar; (**D**) Apparent Density; (**E**) Open Pores and (**F**) Water Absorption over 24 h.

The one-way ANOVA post hoc Tuckey test shows that there was no significant difference in compressive strength and flexural strength between mortar containing EC LWA and FA 6M LWA as the p-value obtained are greater than 0.05 (Figure 6A,B). Compared to the reference sample, a compressive strength reduction of 21% was observed. In the literature, it was found that mortar containing geopolymer mine tailing LWA with replacement rate of 21% for the fraction of 2–4 mm and using cement type II 32.5N had a compressive strength of 25 MPa [5]. With 5% lower replacement rate but higher content of Portland cement (cement type I), the compressive strength of mortar containing FA 6M LWA in this study is much higher compared to the literature results for mortar incorporated with mine tailing fly ash LWA. 

A statistical analysis by one-way ANOVA post hoc Tuckey test showed a significant difference with p value less than 0.05 on the apparent density between EC LWA and FA 6M samples compared to Reference samples (Figure 6D). This result is well correlated with the LWA’s apparent density value (Table 2). Even though FA 6M LWA has a higher porosity compared to EC LWA, FA 6M LWA has a higher apparent density. The small amount of open pores and high amount of closed pores in mortar with EC LWA could be the reason for the results for compressive strength and apparent density (Figure 6C). A similar result was also reported in our previous article, revealing that the compressive strength of mortar incorporating EC LWA had higher compressive strength but lower bulk density [19]. The sintering process during the production of EC LWA, which developed a closed pore system, can be related to the improvement in compressive strength of the resulting mortar [4]. Based on the pore structure characterization through MIP testing, EC LWA mostly contains macropores that could lead to the decrease in apparent density of the resulting mortar compared to the reference mortar (Table 2). The denser ITZ could also be attributed to the strength improvement in EC LWA samples. As LWA aggregate could act as an internal curing agent in the mortar matrix, remaining water in the pore of LWA could react with unhydrated cement and developed denser layer of ITZ between the cement paste and the LWA that could improve the compressive strength of the resulting mortar. This hypothesis was confirmed by the ITZ morphology observation presented further on in this paper.

In FA 6M LWA, high water absorption due to the high amount of open pores is still an issue to be solved. However, when FA 6M LWA was incorporated into a mortar matrix, the resulting mortar did not have such an extremely high-water absorption (Figure 6F). It is also observed that the addition of LWA slightly reduces the flow value of fresh mortar (Figure 6C). The reduction in workability in the EC LWA mortar is an indication that this LWA could absorb the water faster than FA 6M LWA. Even though FA 6M LWA has higher water absorption over 24 h (Table 2), the water seems to penetrate into the pores more slowly. The pore structure of FA 6M LWA that has smaller pore sizes could contribute to this slower rate of water absorption (Figure 4).

### 3.2. Chloride Migration

**Figure 7:**
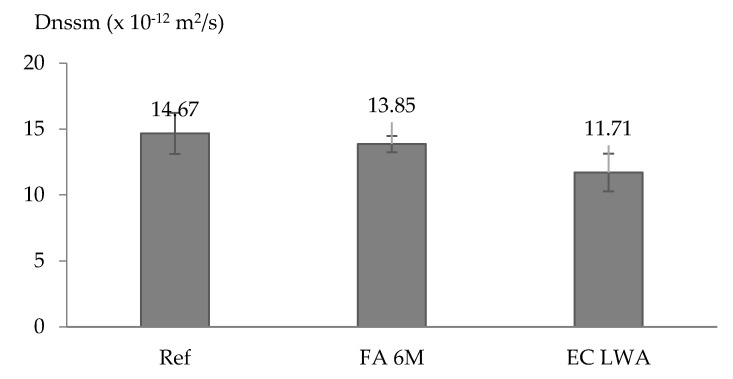
Non steady state chloride migration coefficient of the studied mortar types.

Based on data of the non-steady-state migration coefficient (D_nssm_), incorporating LWA into mortar tends to improve the chloride migration resistance. The reference mortar samples have the highest D_nssm_ value, following by FA 6M samples and EC LWA samples (Figure 7). A statistical analysis by one-way ANOVA showed that only between Ref samples and EC LWA samples a significant difference occurred, while there was no significant difference between Ref samples and FA 6M samples.

It was expected that FA 6M LWA which has high amount of open pores could provide internal curing, making the ITZ denser due to the additional hydration products [13]. However, since this LWA does seem to absorb water less fast than EC LWA; it may be that water absorption is not complete before mortar setting, and part of the additional water could just be considered as mixing water, making the resulting mortar more porous. Hence it could be that with proper water adjustment, the FA 6M mortar sample would have a similar performance regarding chloride migration as the EC LWA mortar sample (instead of being comparable to the reference sample that has no internal curing agent). 

EC LWA with its faster water absorption might be more efficient to induce internal curing in mortar. However, the LWA content is insufficient to provoke a strong effect of internal curing on the chloride migration. In literature, a decrease in chloride migration in mortar by the replacement of 53% of the sand by LWA has been described [13,16]. The effect of higher replacement ratios of EC LWA and FA 6M LWA on chloride migration could be considered a topic for further research. 

### 3.3. Capillary Water Uptake

**Figure 8:**
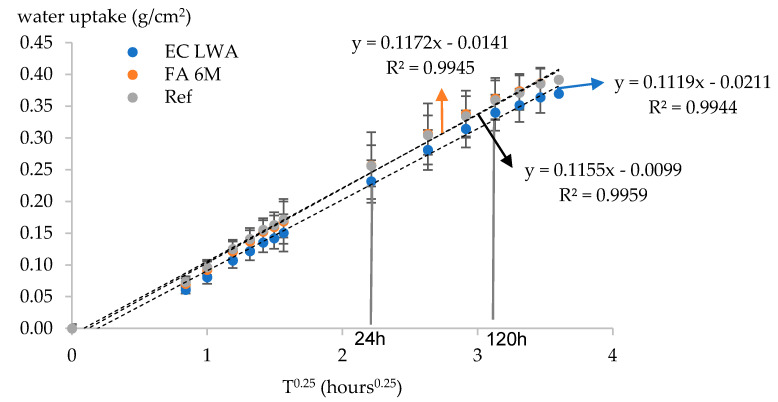
Capillary water uptake of the studied mortar plotted as a function of the fourth root of time. The dashed line refers to linear regression of each series.

In this research we have decided to present the capillary absorption as a function of T^0.25^ instead of T^0.5^ as this recently has been reported to give a better linear correlation [20]. The capillary water uptake was observed over a time period of 168 h. All the data were then used to determine the capillary absorption rate by means of linear regression analysis. The results indeed show that the evolution of capillary water uptake has a linear correlation with T^0.25^, indicated by the R^2^ values of all the series being higher than 0.994 (Figure 8). Similar R^2^ values were also reported in the literature when plotting water uptake as a function of T^0.25^ [20,21,22]. In all series, in the first 24 h, around 0.2 g/cm^2^ water was absorbed by the mortar. This value covered almost 50% of total water uptake by mortar in the total observation period (168 h). It seems that a primary capillary imbibition occurs in this period. From 24 h until 120 h immersion, the rate of water uptake was slightly slower compared to the first 24 h. A slower water uptake could be a first indication that most of the pores are filled with water. From 120 h until the end of the observation period, only small changes in water uptake could be observed. This is an indication that the mortar samples are saturated, and the water penetration into the samples was not driven by capillary force but by diffusion [22].

No significant difference could be observed among all series. The FA 6M series had an almost overlapping capillary uptake pattern with the reference series, while a slight improvement was noticed in the EC LWA series. Similar results were also reported in previous research in which capillary absorption of structural lightweight aggregate concrete was studied [23,24]. It was reported that the usage of LWA did not significantly affect the capillary absorption behavior of the resulting concrete. 

### 3.4. Carbonation Resistance

**Figure 9:**
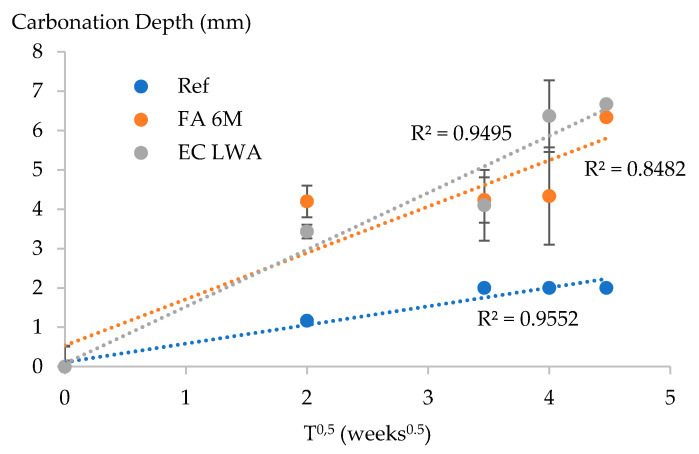
The evolution of the carbonation depth of the studied mortars. The linear regression is presented in dashed line.

**Figure 10:**
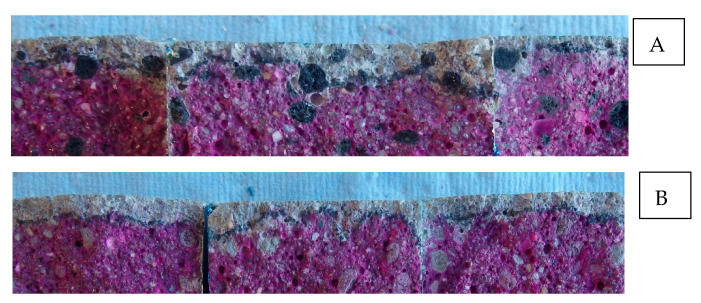
The variability on carbonation depth in (**A**) EC LWA and (**B**) FA 6M LWA mortar samples after being sprayed with phenolphthalein 1%.

No good linear correlation was noticed between the carbonation depth and the square root of time for the mortar samples with LWA (Figure 8). The R^2^ for the linear regression of FA 6M mortar was quite low, followed by EC LWA and reference mortar. It seems that less linearity occurred between the carbonation depth and square root of time for mortar containing FA 6M LWA compared to other mortar types. For the mortar with FA 6M LWA, carbonation depths of the samples that were exposed for 4 weeks showed no significant difference with samples that were exposed for 16 weeks, but at 20 weeks there was a clear increase (Figure 9). In contrast, mortar containing EC LWA showed a significant increase of carbonation depth after being exposed for 16 weeks. After 20 weeks of exposure, similar carbonation depths of around 6.5 mm were observed for the samples with EC LWA and FA 6M LWA. Meanwhile, the carbonation depth for the reference samples was relatively low and showed a lower variability compared to the samples with LWA. High standard deviations were observed for samples with EC and FA 6M LWA. Based on the visual observation of the split sample sprayed with phenolphthalein, it was clear that due to the high porosity of LWA, CO_2_ could penetrate through the LWA causing high variation on the carbonation depth (Figure 10). Similar results have been reported in previous research on using porous Leca aggregate as aggregate substitution in mortar production [23]. 

The rapid increase in carbonation depth for samples containing LWA in the first 4 weeks was mostly due to the high number of open pores in the LWA. This is in agreement with literature, stating that the porosity of LWA has a great influence on the carbonation resistance of its resulting concrete or mortar [23]. At 4 weeks of exposure, samples containing EC LWA have slightly lower carbonation depth compared to the FA 6M sample. This is understandable as EC LWA has more closed pores that might be difficult to be penetrated by CO_2_ compared to FA 6M LWA. However, a significant increase of carbonation depth was observed for the EC LWA sample after being exposed to CO_2_ for 16 weeks. Similar behavior occurs for the FA 6M samples after 20 weeks of exposure. Since Figure 4 shows that the EC LWA only has macro pores, while FA 6M LWA still has around 20% meso pores, it could be reasonably expected that in samples with EC LWA, the significant increase in carbonation depth occurs faster than for the FA 6M samples as CO_2_ will penetrate faster through bigger pores.

In the final weeks of observation, samples containing FA 6M LWA have a slightly better carbonation resistance compared to EC LWA samples. In the FA 6M LWA samples, the excess of sodium hydroxide in the aggregate might react with CO_2_ and form sodium carbonate. Unreacted calcium in fly ash could also react with CO_2_ and form CaCO_3_. It has been also reported in the literature that in fly ash-based alkali activated paste, the C-A-S-H gel will mostly decalcify during accelerated carbonation and lead to the precipitation of CaCO_3_ [25]. Those extra precipitation products could make the matrix around the aggregate denser which can deliver better carbonation resistance. A noticeable improvement in carbonation resistance was also observed when recycled aggregate was incorporated in MSWI bottom ash-based alkali activated systems rather than in control samples (cement based system) [26]. Similar to the observation of carbonated C-A-S-H gel, the enhancing of the carbonation resistance in this system is also due to the decalcification of Ca-bearing phases followed by the precipitation of CaCO_3_, as well as the reaction of Na^+^ with CO_2_ in the pore solution forming sodium carbonates [26].

### 3.5. The Characterization of the Interfacial Transition Zone (ITZ)

**Figure 11:**
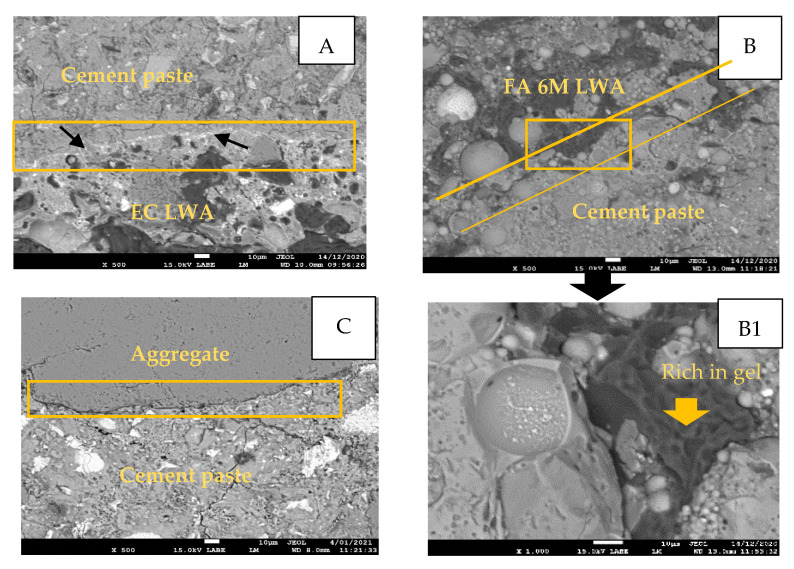
BSE SEM image of the Interfacial Transition Zone of around 10 µm in thickness (**A**) EC LWA; (**B**) FA 6M LWA; and (**C**) normal aggregate with cement paste. (**B1**) magnification of selected area in (**B**) to confirm the presence of gel in the ITZ between FA LWA and cement paste.

The ITZ zone of the EC LWA sample was denser, indicated with the bright whitish line (Figure 11A). In the sample containing FA 6M LWA, the ITZ could not be well observed due to the excess FA on the surface of LWA that might be mixed with cement paste (Figure 11B). On the other hand, a clear gap between aggregate and cement paste was observed in the reference sample (Figure 11C).

The denser ITZ in mortar containing EC LWA is in good agreement with its better performance in chloride migration and capillary water uptake. A dense morphology of the ITZ was also reported for mortar incorporating modified surface slag LWA [27]. The role of LWA as internal curing agent retaining water for release later to react with un-hydrated cement and form hydration products that block the ITZ gap could be clearly noticed for EC LWA. Based on EDS analysis, the amount of calcium and aluminum detected in the ITZ of EC LWA sample was higher compared to the reference samples (Table 4). The ratio between Ca/Si detected in the ITZ of EC samples is 1.1 (Table 4). This ratio is in the range for C-S-H that is usually observed in cement paste, which is between 0.66 to 2.1 [28,29,30]. As the ratio of Ca/Si detected in the ITZ of EC LWA is less than 1.5, the crystal structure of this C-S-H could be jennite [31,32]. Similar patterns were also found in the literature on incorporating slag-based LWA into mortar. Through backscattered electron imaging with the magnification of 1000×, it was found that the calcium rich hydration products in the denser ITZ were C-S-H [27].

In the ITZ between FA 6M LWA and cement paste, pores filled with gel and round unreacted fly ash particles could be sporadically observed (Figure 11B,B1). In literature, clear pictures of gel formation have been taken (with the magnification of 20,000×) in the ITZ of fly ash based geopolymer [33]. The gel form probably was C-A-S-H or N-A-S-H, indicated by the high silica and alumina content in the ITZ (Figure 11B). A high Al/Si ratio (above 0.2) in samples that have lower Ca/Si ratio (lower than 1) was reported to deliver a very weak gel formation of C-A-S-H due to the less reactive alumina in fly ash compared to aluminum in cement clinker [31]. Thus, it is reasonable that a less clear gel formation was observed in the ITZ between FA 6M LWA and cement paste, as the ratio of Ca/Si detected in this area is quite low (below 1), while the Al/Si ratio was high (above 0.2) (Table 4).

## 4. Conclusions

This paper presents the physical properties and durability aspects of mortar containing fly ash-based alkali activated lightweight aggregate. The results show that the properties of hardened mortar containing FA 6M LWA are acceptable compared to a reference without LWA and a mortar with commercial expanded clay (EC) LWA. FA 6M mortar and EC LWA mortar have similar compressive strength, while high water absorption is still an issue that needs to be improved for FA 6M mortar. Exposing the FA 6M LWA to CO_2_ to induce the calcification of unreacted fly ash attached on the surface of LWA is a consideration for further research. With that pre-treatment the open pores on the surface of LWA are expected to be closed, which could decrease the water absorption.

Overall, the FA 6M mortar has comparable durability performance as the reference and the EC LWA mortar sample. FA 6M mortar has a slightly higher chloride migration coefficient, but has slightly better carbonation resistance compared to the other EC LWA sample (differences being insignificant). Moreover, the capillary water uptake performance of FA 6M mortar is similar with reference mortar. A denser ITZ was observed both in EC LWA and FA 6M LWA samples. Summarizing, replacing a limited amount of fine aggregate in mortar with FA 6M LWA could be an alternative solution for increasing the consumption of industrial by-products, as it gives acceptable properties and durability performance.

## Figures and Tables

**Table 1:** Chemical Composition of Fly Ash.

Compound	% by Mass
CaO	3.79
SiO_2_	57.40
Al_2_O_3_	26.17
Fe_2_O_3_	5.99
K_2_O	1.88
MgO	1.43
CuO	0.02
ZnO	0.02
SO_3_	0.98
P_2_O_5_	0.88
TiO_2_	1.13
LOI	0.32

**Table 2:** Physical properties of EC LWA, FA 6M LWA and sand with the size of 2–4 mm.

Test	EC LWA	FA 6M LWA	Sand 2–4 mm
Apparent particle density (AD) (kg/m^3^)	1.25 ± 0.01	2.23 ± 0.01	2.63 ± 0.04
Oven dried density (OD) (kg/m^3^)	0.99 ± 0.01	1.47 ± 0.01	2.48 ± 0.03
SSD particle density (SSD) (kg/m^3^)	1.19 ± 0.01	1.81 ± 0.00	2.54 ± 0.03
Water absorption 24 h (WA_24h_) (%)	20.85 ± 0.09	23.69 ± 0.63	2.19 ± 0.14

**Table 3:** Mix design of three mortar prisms.

Type	LWA OD ^1^ 2–4 (g)	Entrained Water (g)	Sand 2–4 (g)	Sand 0–2 (g)	Cement (g)	Water (g)
Reference	0	0	220	1130	450	225
FA 6M	122	32	0	1196	450	225
EC LWA	82	17	0	1251	450	225

^1^ OD: Oven Dried.

**Table 4:** Resume of EDS mapping analysis on the amount of Ca, Si and Al in ITZ between cement paste and aggregate.

Type	Ca (%)	Si (%)	Al (%)	Ratio Ca/Si	Ratio Al/Si
EC LWA	40.8	36.8	9	1.11	0.24
FA LWA	34	47.3	13.3	0.72	0.28
Ref	32.3	63.3	2	0.51	0.03

## Data Availability

Not applicable.
